# Verification of a Dream after Forty Years

**Published:** 1886-10

**Authors:** 


### *VERIFICATION OF A DREAM AFTER FORTY YEARS.*

The *Detroit Post* was, we believe, the first to publish the following singular statement:

Mr. Notcutt was a highly respectable, independent minister in Ipswitch, the ancestor of a succession of ministers of the same name, in the same town and church. Before he was married the lady to whom he was engaged dreamed that she was, while going over a house which was unknown to her, and in a little room or closet which she had never seen, seized with a violent bleeding from the nose, which could not be suppressed.

Shortly before her marriage the prospective bride paid a visit to her future home with Mr. Notcutt, and seemed to recognize it as one she had seen before. At length, coming upon the little room before mentioned, she exclaimed: "Why, this is the very closet I dreamed I was in when my nose began to bleed!"

The two were married; years passed, the wife became a mother and a grandmother. Exactly forty years was spent in this home, when, one day, while in the identical closet, superintending the disposal of some linen, her nose began to bleed, and continued to do so without intermission. All efforts, as in the dream, which was never forgotten, were unavailing to quench the life-destroying flow, and the good old lady died from its effects.

